# RNA-based approaches in the diagnosis and treatment of obesity

**DOI:** 10.3389/fphar.2026.1882300

**Published:** 2026-08-03

**Authors:** Karishma Parekh, Gitalee Sarker

**Affiliations:** 1 Department of Physiology, Anatomy and Genetics, University of Oxford, Oxford, United Kingdom; 2 Green Templeton College, University of Oxford, Oxford, United Kingdom

**Keywords:** biomarkers, exosomes, non-coding RNAs, obesity, RNA interference

## Abstract

Obesity is a global, complex, and multifactorial disease, and a major risk factor for diabetes, cardiovascular disease, fatty liver disease, and cancer. Despite its high prevalence, effective diagnostic and therapeutic strategies remain limited. Conventional anthropometric measurements, such as body mass index (BMI) and waist circumference, offer limited accuracy in assessing the impact of excess adiposity and related comorbidities on individual patients. Current anti-obesity pharmacotherapies also show inconsistent efficacy, are often associated with systemic side effects, and poor adherence to necessary lifestyle modifications, which limit their long-term success. Emerging evidence underscores the critical role of non-coding RNAs, including microRNAs, long non-coding RNAs, circular RNAs, and tRNA-derived small RNAs in key metabolic processes such as adipogenesis, insulin signalling, and appetite control. Notably, the profiles of many circulating miRNAs have been reported to differ between patients with obesity compared to healthy lean individuals, suggesting their potential as minimally invasive diagnostic biomarkers. In this mini-review, we discuss the clinical promise of RNA-based approaches for disease risk assessment and as therapeutic targets in obesity. In addition, we discuss key challenges, including biomarker validation and effective RNA delivery systems to the target organs. Multi-omics approaches may help overcome many of these limitations and enable the development of targeted and personalized RNA-based anti-obesity therapies.

## 1 Introduction

Obesity is a chronic disease of energy imbalance, arising from a combination of environmental, genetic, and epigenetic factors (Lin and Li, 2021). It has evolved into a major health crisis, predisposing 1 in 8 people worldwide to a higher risk of cancer, cardiovascular disease, type 2 diabetes (T2DM), and metabolic dysfunction-associated steatotic liver disease (MASLD) (Blüher, 2019). Despite its prevalence, developing effective obesity therapies has been a hurdle in medical research. Dietary and lifestyle changes are foundational to obesity management but are limited by poor long-term adherence and high relapse rates.

Recent promise has been shown by glucagon-like peptide 1 (GLP-1) receptor agonists, such as semaglutide (Wilding et al., 2021), and by dual GLP-1 and glucose-dependent insulinotropic polypeptide (GIP) receptor agonists, such as tirzepatide (Jastreboff et al., 2022). These lead to significant weight loss by reducing energy intake through appetite suppression, delayed gastric emptying, and enhanced central satiety signalling. However, there are caveats to their use, such as uncertainty of whether the metabolic changes induced by these drugs sustain weight loss over time without requiring lifelong use (West et al., 2026). Importantly, discontinuation of such drugs is frequently associated with substantial weight regain (West et al., 2026; Ceriello et al., 2026). In addition, these anti-obesity drugs preferentially target energy intake rather than the underlying pathophysiological drivers of obesity.

These limitations emphasise the need for novel therapeutic strategies that target the molecular drivers of obesity. Over the past 2 decades, non-coding RNAs (ncRNAs) have emerged as critical regulators within genetic networks governing healthy metabolic functions (Chen and Kim, 2024). They play key roles in processes such as adipogenesis, lipid metabolism, insulin signalling, and inflammatory responses-all of which are central to obesity development and progression (Dandare et al., 2023). Alterations in the ncRNAs’ expression have been increasingly linked to obesity and its associated comorbidities, positioning these molecules as promising biomarkers and potential therapeutic targets.

## 2 RNA types involved in metabolic health and obesity

### 2.1 miRNAs

**FIGURE 1:**
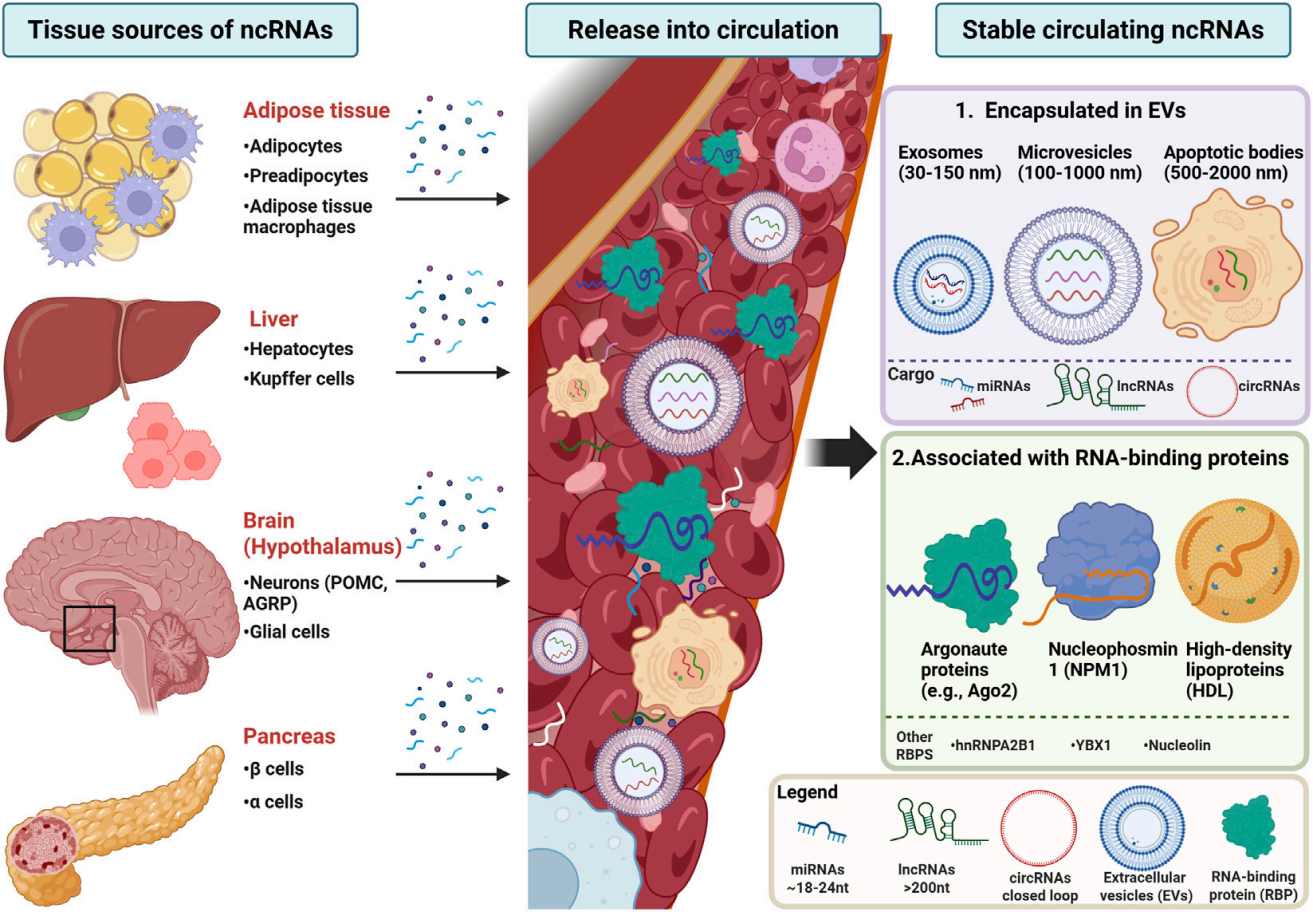
Origin, circulation and functional role of non-coding RNAs in obesity. Non-coding RNAs (ncRNAs), including microRNAs (miRNAs), long non-coding RNAs (lncRNAs), and circular RNAs (circRNAs), are released from multiple metabolically active tissues and enter the circulation where they act as stable intercellular signalling molecules. Major tissue sources include adipose tissue, liver, hypothalamic region of the brain, pancreas, and muscle. These ncRNAs are released into the bloodstream, facilitating systemic communication between organs. In circulation, ncRNAs are protected from degradation through two principal mechanisms: (1) encapsulation within extracellular vesicles (EVs), such as exosomes, microvesicles, and apoptotic bodies, which transport RNA cargo between cells; (2) associated with RNA-binding proteins, including Argonaute proteins (e.g., Ago2), nucleophosmin 1 (NPM1), and lipoprotein complexes such as high-density lipoproteins (HDL). These stabilisation strategies enable ncRNAs to persist in extracellular environments and maintain biological activity. Circulating ncRNAs function as endocrine-like mediators of intercellular communication and play key roles in regulating metabolic homeostasis, adipogenesis and lipid metabolism, immune and inflammatory responses, stress adaptation, and neuronal control of appetite and energy balance. Dysregulation of these signalling networks is implicated in the development and progression of obesity and its associated comorbidities. Created with BioRender.com.

microRNAs (miRNAs) are small non-coding RNA molecules (∼18-24 nucleotides) that mediate post-transcriptional gene regulation (Cai et al., 2009). They function primarily in the cytoplasm within RNA-induced silencing complexes (RISCs), targeting messenger RNA (mRNA) for degradation or translational repression (Iwakawa and Tomari, 2022). miRNAs also circulate in body fluids, protected within vesicles such as exosomes (Valadi et al., 2007), or bound to RNA-binding proteins like Argonautes (O’Brien et al., 2018), enabling intercellular communication to coordinate gene regulation across tissues involved in malignancy (Peng and Croce, 2016; Klein et al., 2010), immunity (O’Connell et al., 2010), stress adaptation (Leung and Sharp, 2010), and metabolic homeostasis (Najafi-Shoushtari et al., 2010; Trajkovski et al., 2011) (Figure 1).

Adipogenesis is an essential physiological process to produce adipocytes, the primary cells responsible for storing energy in the form of triacylglycerols (Ali et al., 2013). This process is tightly regulated by a network of transcription factors and ncRNAs, particularly miRNAs (Lee et al., 2019; Hilton et al., 2013). For instance, miR-6402 inhibits the BMP4/BMPR2 signalling pathway, resulting in reduced adipogenesis in both 3T3-L1 adipocytes and the adipose tissue of diet-induced obese (DIO) mice (Elsheikh et al., 2025). miR-30a promotes adipogenesis and white-to-beige fat conversion by enhancing PPARγ activity, a “master” regulator of adipocyte differentiation (Saha et al., 2020). Mechanistically, miR-30a suppresses SIRT-1, a negative regulator of PPARγ, and its pathways, thereby amplifying PPARγ signalling (Mayoral et al., 2015; Majeed et al., 2021; Cui et al., 2018). In contrast, the miR-301b/miR-130b cluster suppresses PPARγ expression, reducing adipogenic capacity in mesenchymal stem cells derived from adipose tissue, umbilical cord, and bone marrow (Liu et al., 2017), possibly via AMPK inhibition (Luo et al., 2022).

In addition to PPARγ-centered regulation, AMPK signalling also plays a complex role in energy homeostasis in a context-dependent manner (Hardie et al., 2012). In the hypothalamus, pro-opiomelanocortin (POMC) neuron-specific deletion of AMPK results in obese mice due to reduced energy expenditure and disrupted food intake, whereas AMPK deletion in agouti-related peptide (AgRP) neurons produces a lean phenotype with increased melanocortin sensitivity (Claret et al., 2007). This highlights the limitation of global manipulation of hypothalamus-wide AMPK signalling. A recent study supports a cell-type-specific role for miRNAs in this context (Price et al., 2024). Selective knockout (KO) of miR-33 in AgRP neurons increases food consumption and metabolic dysfunction in DIO mice, while POMC-specific miR-33 KO shows minimal effects, suggesting therapeutic potential in cell-type-specific actions of miRNA targeting.

Beyond intracellular roles, miRNAs can also operate as endocrine-like signals. miRNAs can be packaged into small extracellular vesicles (sEVs) such as exosomes, enabling inter-organ communication (Brandao et al., 2022). Adipose tissue has emerged as a major contributor to the circulating miRNA pool (Huang and Xu, 2021), as shown by reduced circulating miRNA levels in adipocyte-specific Dicer KO (ADicerKO) models (Thomou et al., 2017). These exosomal miRNAs mediate inter-organ cross-talk. For instance, loss of adipose-derived miRNAs increases hepatic FGF21 activity, while suppression of hepatic FGF21 requires exosomal delivery of miR-99b (Thomou et al., 2017). Similarly, Lino et al. reported that insulin stimulation of adipocytes alters sEV miRNA content involved in hepatic metabolism, including Let-7, miR-103-3p, and miR-3075 (Lino et al., 2024; Ji et al., 2021). Adipose-derived exosomes also influence pancreatic β-cells, where depletion of miR-138-5p improves insulin sensitivity and cell survival (Fan and Li, 2025).

Adipose tissue macrophages (ATMs) further contribute to this signalling network. Exosomes from obese ATMs impair insulin sensitivity in adipocytes, myocytes, and hepatocytes, largely via elevated miR-155 levels (Ying et al., 2017). However, global miR-155 KO only partially rescued diet-induced insulin resistance and glucose intolerance, suggesting the involvement of additional miRNAs. Indeed, selective depletion of miR-29a from obese ATM-derived exosomes reduces their capacity to induce insulin resistance *in vivo* (Liu et al., 2019), supporting a multi-miRNA regulatory model rather than single-miRNA effects.

### 2.2 lncRNAs

Long non-coding (lnc) RNAs (>200 nucleotides) regulate gene expression through diverse mechanisms (Tsagakis et al., 2020). They may influence histone modification (Jiang et al., 2022), act as miRNA sponges (Xiao et al., 2020), directly bind to mRNA to affect its stability and translation (Carrieri et al., 2012). They also regulate transcription through interaction with DNA-binding proteins (Huarte et al., 2010) and can affect alternative mRNA splicing (Tripathi et al., 2010) and post-transcriptional protein modification (Wang C. et al., 2020) (Figure 1). Similar to miRNAs, lncRNAs’ regulatory functions are often conditional on cell type and environment (Statello et al., 2021). Advances in sequencing technologies have identified thousands of lncRNAs with important roles in metabolic signalling networks (Uszczynska-Ratajczak et al., 2018; Zhao and Lin, 2015).

Several lncRNAs have been implicated in adipose tissue function. For instance, Lnc-U90926 is highly expressed in white adipose tissue but is reduced in obese models such as DIO, *ob*/*ob*, and *db*/*db* mice (Chen et al., 2017). Using lentivirus-mediated knockdown and overexpression in 3T3-L1 preadipocytes, Lnc-U90926 was shown to be pro-adipogenic. The results indicated that Lnc-U90926 can reduce the transactivation of the adipose-specific PPARγ2 promoter (Fajas et al., 1997). However, given that Lnc-U90926 localises to the cytoplasm while PPARγ2 functions in the nucleus, a direct interaction is unlikely, suggesting that further investigation is required to elucidate the underlying mechanism.

Beyond adipogenesis, lncRNAs also regulate lipid metabolism. LINC00473 and Hnscr promote adipocyte lipolysis through modulation of the cAMP/PKA signalling, a central regulatory pathway of lipid metabolism (Tran et al., 2020; Guo et al., 2023). Similarly, the human-specific MEK6-AS1 has been linked to adipogenesis, adipocyte differentiation, fatty acid metabolism, and osteogenic differentiation (Li D. et al., 2025). Notably, the functional characterisation of MEK6-AS1 relies on an *in vitro* ectopic adipogenesis model, as no rodent orthologue exists, limiting the insight into systemic effects from *in vivo* genetic manipulation.

Many lncRNAs also play important roles in hepatic metabolism. For example, Blnc1 promotes brown and beige adipocyte differentiation (Zhao et al., 2014), yet in hepatocytes, it facilitates pathological lipogenesis through interaction with EDF1, linking it to MASLD (Zhao et al., 2018). In addition, Hedgehog signalling has been shown to induce the lncRNA Hilnc, which interacts with the RNA-binding protein IGF2BP2 to enhance the stability of PPARγ mRNA (Jiang et al., 2021). However, the human homologue of Hilnc has only been identified based on functional similarity rather than sequence conservation.

Additionally, *in vivo* studies reinforce the tissue-specific actions of lncRNAs. Global deletion of Nron (NronKO) in DIO mice reduces adiposity, adipose inflammation, and hepatic steatosis, alongside decreased hepatic FGF21 levels (Liu B. et al., 2023). Accordingly, hepatic knockdown of FGF21 in NronKO mice restored liver fat accumulation, but the reduced body weight remained unchanged. These findings emphasise the tissue-specific roles of lncRNAs in metabolic regulation and flag the need to consider potential off-target effects when manipulating a single lncRNA.

### 2.3 circRNAs

Circular RNAs (circRNAs) are covalently closed ncRNAs, best known for acting as miRNA sponges, that reduce miRNA-mediated repression of target mRNAs (Panda, 2018). Several circRNAs have been implicated in adipogenesis through this mechanism. For example, circSAMD4A has been shown to sponge miR-138-5p and miR-127 in cultured human and porcine preadipocytes, respectively (Liu et al., 2020; Liu et al., 2025). Similarly, circFLT1 has been shown to promote adipocyte differentiation and suppress proliferation in bovine adipocytes, which may be mediated by sponging miR-93 (Kang et al., 2020). Another miRNA sponge identified in bovine preadipocytes is circBDP1, which promotes proliferation and differentiation, likely by competitively adsorbing miR-181b and miR-204 as suggested by dual-luciferase reporter reactions (Zhang et al., 2022). Apart from miRNA sponging, circRNAs can also regulate adipogenesis through interactions with RNA-binding proteins. For instance, knockdown of hsa_circH19 in human adipose-derived stem cells led to increased lipid droplet formation and upregulation of adipogenic genes, including C/EBPα, PPARγ and SREBP1c, an effect linked to its interaction with polypyrimidine tract-binding protein 1 (PTBP1) (Zhu et al., 2020).

circRNAs are also implicated in metabolic disease, particularly in the regulation of hepatic lipid accumulation and pancreatic β-cell function. Reduced expression of circRNA_0001805 has been observed in both *in vitro* models of hepatic steatosis and murine models of MASLD, suggesting a potential association with disease progression (Li et al., 2021). This observation is further supported by significantly lower levels of circRNA_0001805 measured in liver tissues from MASLD patients, although the small human cohort size and limited clinical characterisation restrict interpretation. In pancreatic β-cells, circGlis3 appears to play a compensatory role during early metabolic stress by promoting insulin secretion and β-cell survival (Liu Y. et al., 2023). Mechanistically, circGlis3 acts as a competing endogenous RNA, sponging miR-124-3p, thereby relieving repression of Creb1 and NeuroD1, key transcription factors involved in β-cell function and insulin gene expression. Additionally, overexpression of circGlis3 preserves β-cell mass and reduces apoptosis in *db/db* mice and similarly restricts apoptosis in bovine intramuscular preadipocytes (Yu et al., 2026). However, its decline in later disease stages, along with reliance on overexpression models, raises concerns about physiological relevance.

### 2.4 tsRNAs: tRFs and tiRNAs

tRNA-derived stress-induced RNAs (tiRNAs), also referred to as tRNA halves, and tRNA-derived fragments (tRFs) are both tRNA-derived small RNAs (tsRNAs) generated by precise enzymatic cleavage of mature or precursor transfer RNAs (Jiang and Yan, 2019). Interest in tsRNAs initially emerged from studies of intergenerational metabolic inheritance. They are abundant in sperm, and a paternal high-fat diet can alter sperm tsRNA profiles and RNA modification patterns (Chen et al., 2016). Notably, injection of these fragments alone into zygotes is sufficient to induce glucose intolerance in offspring, with effects traced to altered embryonic and islet gene expression (Chen et al., 2016). Sarker et al. extended this concept by showing that this germline reprogramming can also be initiated indirectly, one generation earlier, by maternal high-fat diet during pregnancy, which alters the sperm tsRNA profile of the resulting sons and, upon zygote injection, transmits obesity to the next-generation (Sarker et al., 2019).

More recent work has implicated tsRNAs directly in adipose biology. In adipose tissue itself, tRFs derived from tRNA-Gly-GCC and related isoforms are differentially expressed between lean and obese states across pigs and mice, though interpretation is limited by cross-species differences, including the complete absence of brown adipose tissue in pigs (Liao et al., 2023). Building on these observations, Lei et al. identified tRF-Gly-GCC-037 as a conserved, abundant tsRNA that is elevated in obese pig and mouse adipose tissue and promotes adipogenesis and diet-induced obesity, by associating with AGO3 to silence Rac1 (Lei et al., 2025). While Rac1 rescue experiments provide causal support, the AGO3–Rac1 interaction lacks direct structural validation, and the *in vivo* delivery strategy does not distinguish systemic from depot-specific effects.

Evidence in humans remains limited. Zhang et al. reported that tsRNAs are dysregulated in the adipose tissue of overweight individuals with T2DM and are linked to AMPK and insulin-signalling pathways (Zhang J. et al., 2023). However, because the study did not include non-diabetic controls, it remains unclear whether these changes are attributable to obesity, diabetes, or both.

### 2.5 piRNAs

PIWI-interacting RNAs (piRNAs) are 24–31 nucleotide single-stranded small RNAs that bind PIWI, a class of Argonaute proteins, and were classically considered germline-restricted, where they silence transposable elements through the self-amplifying ping-pong cycle (Ozata et al., 2019). Although PIWI protein structure and this silencing mechanism are well conserved across animals, piRNA sequences themselves show markedly low cross-species conservation, and somatic PIWI/piRNA activity has so far been linked mainly to cancer, cardiovascular, and neurodegenerative phenotypes rather than metabolic disease specifically (Patel et al., 2024).

Evidence for a metabolic role outside the gonad first emerged from *Drosophila*, where the fat body, a functional analogue of mammalian liver and adipose tissue, was shown to host an intact piRNA pathway with a transposon-dependent mechanism (Jones et al., 2016). Importantly, disruption of this pathway reduced lipid storage and impaired metabolism, indicating that piRNAs contribute to energy homeostasis. In locusts, Wang et al. extended piRNA function beyond transposon silencing, showing that brain-expressed Piwi1 and intronic piRNAs promote splicing of neuropeptide F1 (NPF1) pre-mRNA, and Piwi1 knockdown reduces food intake and body weight in a manner rescuable by synthetic NPF1, identifying a splicing-based rather than ping-pong-based mode of metabolic regulation (Wang H. et al., 2022).

Unfortunately, there is little direct human evidence. Donkin et al. reported that piRNA content of sperm differs between lean and obese men and is remodelled after bariatric surgery, with differentially expressed piRNAs predicted to target chromatin- and CART-related genes relevant to appetite control, though this remains a germline, bioinformatically-predicted association rather than functional evidence (Donkin et al., 2016). Causal evidence for piRNA-dependent metabolic regulation is therefore currently strongest in invertebrate models, leaving the direct relevance of piRNAs to human adipose or hepatic metabolism questionable.

### 2.6 snoRNAs

Small nucleolar RNAs (snoRNAs) are a class of non-coding RNAs, typically 60-300 nucleotides long, that accumulate in the nucleolus. snoRNAs were classically known for guiding chemical modification of ribosomal and other RNAs but are now recognised as important regulators of metabolic processes. Evidence for their role in obesity is mixed: He et al. reported that SNORD126 expression was reduced in the fat tissue of high fat diet-fed rats, although AAV-mediated SNORD126 overexpression in fat pads promoted adipogenesis in lean rats (He et al., 2021). In contrast, Zhang et al. showed that SNORD46 is elevated in obese human serum and promotes obesity by sequestering interleukin-15, thereby blocking its lipolytic and immune-activating functions (Zhang Y. et al., 2023). Importantly, a SNORD46 power-inhibitor can reverse obesity and restore anti-tumour immunity in mice, highlighting its potential as a therapeutic target.

## 3 RNA as diagnostic biomarkers–early diagnosis, risk stratification, comorbidities

The presence of miRNAs as stable circulating molecules has created interest in their use as detectable, non-invasive, blood-based markers of metabolic health and disease (Condrat et al., 2020). However, the clinical translation of circulating RNA biomarkers depends critically on distinguishing whether a circulating miRNA is being proposed as a diagnostic, predictive, prognostic, or pharmacodynamic biomarker. Much of the existing obesity-RNA literature blurs these categories, and few studies test whether a candidate RNA adds predictive value beyond established measures such as BMI, waist circumference (Condrat et al., 2020; Sommer et al., 2020), HOMA-IR (Fouad et al., 2025), lipid profile (Kawamoto et al., 2011), or liver fat (Ducluzeau et al., 2013).

Several miRNAs have been proposed as diagnostic markers in obesity-related metabolic disease. Catanzaro et al. identified elevated miR-155-5p in obese patients with T2DM relative to obese patients without T2DM and showed that combining miR-155-5p with IL-8, leptin, and RAGE improved discrimination of diabetes risk (*R* ^2^ = 0.7) beyond the miRNA alone (Catanzaro et al., 2023). Similarly, Masoumi-Ardakani et al. reported elevated serum miR-33b in both hypercholesterolaemic and obese cohorts, with modest discriminative performance (AUC 0.74–0.76) that did not exceed conventional lipid markers such as total cholesterol or LDL-cholesterol (Masoumi-Ardakani et al., 2025). Both studies, however, used small cohorts and did not formally test whether the RNA signature improved prediction beyond variables routinely available at the bedside.

A separate, more rigorous case is the prediction of long-term treatment outcomes. Mela et al. investigated whether preoperative serum miRNAs could predict durable weight loss 5–8 years after bariatric surgery, and identified miR-365b-5p and miR-222-5p as combined predictors with an AUC of 0.874 that substantially exceeded either marker alone (Mela et al., 2025). By forecasting a future treatment-specific outcome, this study represents a genuine predictive-rather than purely diagnostic-biomarker. However, diet and physical activity during the multi-year follow-up were not captured, leaving the possibility that lifestyle factors may have driven some of the observed variance in outcomes.

On the other hand, Alkandari et al. reported circulating miRNAs could track the biological response to Roux-en-Y gastric bypass surgery, rather than predicting its outcome in advance (Alkandari et al., 2018). Over 12 months following the surgery, 48 miRNAs were significantly altered and several correlated with BMI, weight loss, and fasting glucose level. Similarly, miR-92a was found to be elevated in severe obesity and associated with hyperinsulinaemia, HbA1c, and HOMA-IR declined to near-control levels 6 months after bariatric surgery (Cereijo et al., 2020). This suggests a possible function of circulating miRNAs as pharmacodynamic markers following weight-loss interventions.

One of the most extensively studied circulating miRNAs is miR-122, but its role as an obesity biomarker remains uncertain. miR-122 is a liver-enriched miRNA whose circulating levels closely track hepatic fat content and conventional liver enzymes rather than adiposity *per se*. Rega-Kaun et al. observed that miR-122 levels reduced after Roux-en-Y gastric bypass, consistent with improved hepatic status, but did not show that it added predictive information beyond routine liver markers such as ALT and γGT (Rega-Kaun et al., 2020). In contrast, the CENTRAL trial found that a greater rise in miR-122 over 18 months was associated with less reduction in hepatic fat, though this relationship was influenced by baseline glucose levels (Wang M. et al., 2022). Together, these discordant findings suggest that miR-122 levels may depend heavily on intervention type and metabolic status, limiting its value as a standalone obesity biomarker.

LncRNAs are also being investigated as biomarkers of obesity. Liu et al. identified 1268 differentially expressed lncRNAs in the adipose tissue from prepubertal children with obesity, with lncRNA RP11-20G13.3 showing positive correlation with BMI-SDS, waist circumference, fasting insulin, LDL-cholesterol, and leptin (Liu et al., 2018). Though a cross-sectional study, it makes comparisons against routine clinical parameters that obesity RNA-biomarker studies have generally lacked. However, the study cohort was very small, and several proposed correlations with insulin resistance and lipid indices did not reach significance. Further longitudinal investigations are needed to discover whether RP11-20G13.3 predicts future metabolic trajectory, comorbidity progression, or response to intervention.

Among circRNAs, circSAMD4A has emerged as a promising obesity biomarker. In the visceral adipose tissue of obese individuals, circSAMD4A was overexpressed and associated with non-remission 1 year after bariatric surgery (Liu et al., 2020). The authors reported high predictive accuracy (AUC 0.944) and provided strong mechanistic evidence that a circSAMD4A–miR-138-5p–EZH2 axis promotes adipogenesis in both human preadipocytes and obese murine models. However, the diagnostic accuracy metrics were derived and tested within the same single-centre cohort, with no independent validation, raising concerns that the reported performance is over-optimistic. In addition, circSAMD4A was not benchmarked against baseline BMI, diabetes status, or other clinical predictors of surgical non-remission, so its incremental value remains unproven. Finally, as it predicts response to bariatric surgery rather than disease progression itself, circSAMD4A is more accurately described as a predictive rather than a prognostic biomarker.

The biomarker potential of piRNA and snoRNA in obesity remains largely unexplored, with most studies centred on cancer rather than metabolic disease (Ahmadi et al., 2024; Huang et al., 2022). A small number of studies have linked these RNAs with obesity-associated comorbidities: piR-019825 and several snoRNAs are depleted in diabetic kidney disease (Hart et al., 2024), hsa-piR-020009 and hsa-piR-006426 are downregulated in heart failure (Yang et al., 2018) and SNORD113.2 rises acutely following ST elevation myocardial infarction (Håkansson et al., 2019). However, these disorders arise from multiple interacting genetic, metabolic and environmental factors, making it difficult to determine whether these RNA signatures reflect obesity. As a result, the evidence supporting piRNAs and snoRNAs as diagnostic, prognostic or risk-stratification markers for obesity remains sparse and largely indirect.

Unlike piRNAs and snoRNAs, tsRNAs have been studied directly within obesity-related metabolic disease, though the evidence for their clinical utility remains at an early stage. Zhang et al. identified several dysregulated adipose-derived tsRNAs (e.g., tRF-1-28-Glu-TTC-3-M2, tRF-1-31-His-GTG-1) in overweight individuals with T2DM (Zhang Y et al., 2023). However, the small sample size and reliance on adipose tissue profiling limit the study’s relevance to biomarker discovery. More translational relevance comes from Huang et al. who demonstrated that circulating tRF-Val-CAC-005, tRF-Ala-CGC-006, and tiRNA-His-GTG-001 could distinguish MASLD patients from controls with good diagnostic accuracy and correlated with fibrosis severity (Huang et al., 2021). Though these findings suggest potential risk-stratification in obesity-associated liver disease, the cross-sectional design means that their predictive value has yet to be determined.

A recurring weakness across all RNA classes reviewed here is that studies fail to compare candidates against established clinical measures, leaving their added clinical value unproven. Much of the evidence is also tissue-based, limiting translational capacity, and biomarker claims are frequently overstated, as illustrated by the reclassification of circSAMD4A (Liu et al., 2020) from prognostic to predictive biomarker. Importantly, the expansion of biomarker discovery beyond miRNAs to include tsRNAs, piRNAs, and snoRNAs has not addressed the field’s core challenges: small cohort sizes, lack of independent validation, methodological heterogeneity, and limited assessment of clinical utility. Future studies should prioritise rigorous prospective validation of the most promising candidates, rather than continued discovery of new ones.

## 4 RNA-based therapeutics

The central challenge in RNA-based obesity therapeutics is delivery rather than target discovery. The literature is explored here along the trajectory of target identification to RNA cargo design, delivery engineering, and precision targeting.

**FIGURE 2:**
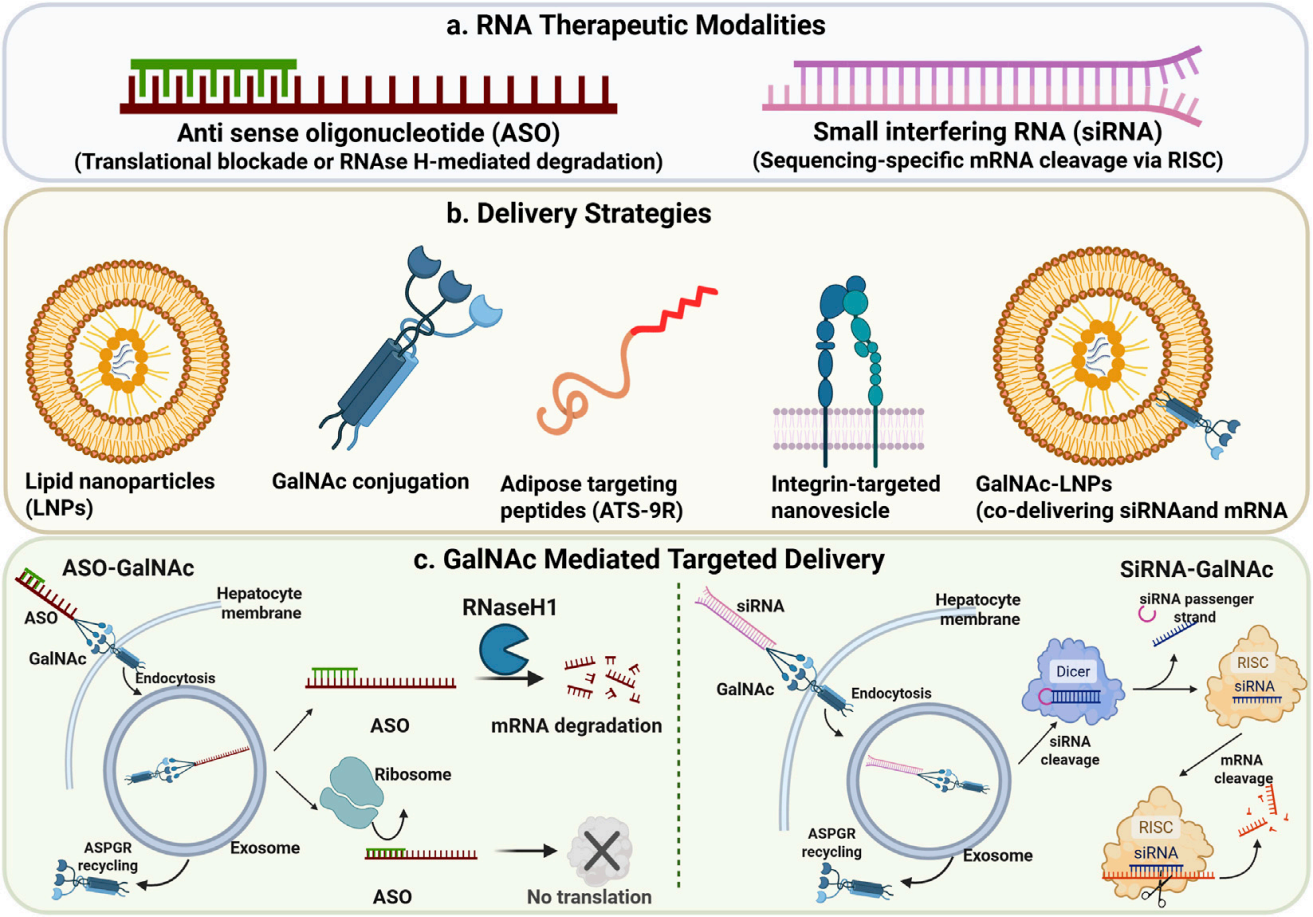
RNA-based therapeutic strategies. **(a)** RNA-based therapeutics include antisense oligonucleotides (ASOs) and small interfering RNAs (siRNAs), which function through two distinct mechanisms. ASOs suppress gene expression either by sterically blocking translation or by RNase H1-mediated mRNA degradation. siRNAs guide sequence-specific cleavage of complementary mRNA via the Dicer-RISC complex. **(b)** Efficient delivery of RNA therapeutics can be achieved through different approaches. Lipid nanoparticles (LNPs) encapsulate RNA cargo to facilitate cellular uptake. GalNAc conjugation ensures targeted delivery via binding to asialoglycoprotein receptors (ASGPR). Adipose tissue-targeting peptides, integrin-targeted nanovesicles, and ligand-directed LNPs are also capable of delivering RNA cargo. **(c)** GalNAc-mediated delivery of ASOs and siRNAs to hepatocytes. Created with BioRender.com.

Small interfering RNAs (siRNAs) are a class of double-stranded RNA molecules that can silence genes by guiding RISC to cleave complementary mRNAs (Neumeier and Meister, 2021), whereas antisense oligonucleotides (ASOs) inhibit gene expression either by physically blocking translation or by recruiting RNase H1 to degrade the target transcript (Lauffer et al., 2024) (Figure 2). While both methods are mechanistically mature, their clinical success has largely depended on tissue-specific delivery. The most successful example is GalNAc-ASGPR conjugation: N-acetylgalactosamine (GalNAc), which binds to the asialoglycoprotein receptor (ASGPR) with high affinity, a receptor expressed almost exclusively on liver hepatocytes. By attaching GalNAc to an siRNA or ASO, the drug is efficiently routed into the liver, which can be harnessed for targeted treatment of obesity-associated diseases like dyslipidaemia and chronic liver disease (Ray et al., 2025; Sanyal et al., 2025). However, the success of the GalNAc-ASGPR system has proven difficult to replicate outside the liver.

Most adipose-targeting strategies rely on receptors that are enriched in fat rather than uniquely expressed, raising concerns about off-target delivery. For instance, Yong et al. conjugated a prohibitin-binding peptide to oligoarginine to deliver TACE-silencing short hairpin RNA (shRNA) to visceral adipose tissue macrophages, improving insulin sensitivity in obese mice (Yong et al., 2017). However, prohibitin is widely expressed (Signorile et al., 2019), and the apparent targeting may reflect local accessibility following intraperitoneal injection, rather than any genuine adipose specificity. Similarly, Vasdev et al. targeted integrin αvβ3 using cyclic RGD-decorated lipid nanovesicles co-loaded with a dendrimer-miR-130b complex (Vasdev et al., 2025). However, αvβ3 is broadly expressed on angiogenic endothelium and is particularly abundant in tumours (Zhang M. et al., 2023), creating substantial potential for off-target accumulation. Moreover, the study included only three animals per group, lacked off-target biodistribution analysis, and assessed results over just 28 days. Although these studies demonstrate that adipose tissue silencing is achievable in principle, they fall short of establishing a reproducible or clinically scalable platform. Neither approach has been independently replicated, and both used unencapsulated or poorly scalable formulations rather than a clinically viable nanoparticle.

Lipid nanoparticles (LNPs) are a delivery vehicle for cargo, encapsulating nucleic acids within an ionisable lipid shell to protect them from degradation and enable endosomal escape. Their capacity to carry larger or more diverse payloads than conjugates underlie their already-validated clinical use, from patisiran in hereditary transthyretin amyloidosis (Adams et al., 2018) to mRNA COVID-19 vaccines (Wilson and Geetha, 2022). Stewart et al. interestingly combined the GalNAc targeting technique with the LNP delivery method to co-deliver a large cargo of IL-27 mRNA and DPP4 siRNA with hepatocyte specificity, achieving greater weight loss than either nucleic acid alone (Stewart et al., 2025). However, achieving adipose selectivity with an LNP remains an unsolved challenge. Greco et al. addressed this more systematically than previous studies, screening 649 LNP formulations across adipocytes and macrophages in parallel, and using machine learning to identify lipid compositions that favoured adipocyte uptake (Greco et al., 2025). Yet even their optimised formulation showed substantial macrophage transfection, and the validation depended on local fat pad injection rather than systemic delivery. Moreover, the approach was never tested in visceral adipose tissue, the depot most strongly linked to metabolic disease. Bartesaghi et al.'s FGF21-miRNA-LNP sidesteps this problem rather than solving it: FGF21 is a secreted hormone, so the protein acts systemically once translated, such that the LNP never needed to reach adipose tissue specifically to produce a metabolic effect (Bartesaghi et al., 2022).

Lei et al. took yet another route, exploiting organ anatomy rather than any receptor-mediated targeting (Le et al., 2026). Their AH-LNP absorbs peritoneal proteins after intraperitoneal injection and expands in size, preventing entry into most abdominal organs, yet passing freely into the pancreas, which uniquely lacks a VLDL receptor. This platform achieved durable (∼90% at 180 days) MHC-I knockdown in a diabetic mouse model, demonstrating that physical exclusion may substitute for receptor specificity, which is difficult to achieve in other tissues such as adipose tissue. However, the study’s relevance to obesity is limited. Importantly, the particles preferentially transfect stromal and fibroblast populations rather than insulin-secreting β-cells, and the approach depends on anatomical features that vary substantially across species. Consequently, it remains unclear whether this size-exclusion principle will translate effectively to humans.

Engineered extracellular vesicles (EVs), particularly exosomes, offer an alternative approach to tissue targeting. Unlike LNPs, which can be limited by complex manufacturing, suboptimal stability, and immunogenicity, exosomes are endogenous carriers of intercellular communication and therefore possess low immunogenicity, favourable biocompatibility, and the ability to transport diverse cargo across biological barriers. Guo et al. demonstrate this with an ultrasound-responsive exosome platform in which a detachable PEG coating limited off-target uptake and prolonged circulation, while focused ultrasound triggered local release of Bmp7 mRNA within omental adipose tissue, producing white fat browning, increased Ucp1 expression, and weight loss in obese mice (Guo et al., 2021). Conceptually, this resembles Lei et al.'s strategy: a non-receptor selectivity mechanism, here an external trigger rather than anatomical exclusion, substitutes for the adipose receptor that does not exist (Le et al., 2026; Guo et al., 2021). However, EV-based systems face their own translational barriers. EV populations are inherently heterogeneous; large-scale production and cargo loading are not yet standardised; and different isolation methods can yield vesicles with distinct biological properties, so batch-to-batch reproducibility remains a challenge.

While delivery remains the principal translational bottleneck, expanding the range of therapeutic RNA cargoes has become a parallel area of investigation. However, the evidence supporting these newer RNA classes (miRNA mimics/antagomirs, circRNAs, tRFs, and aptamers) is generally less developed than that for siRNAs and ASOs.

miRNAs are particularly attractive because, unlike an siRNA, which silences a single transcript, a miRNA regulates an entire gene network, so restoring a depleted protective miRNA (a mimic) or inhibiting an overactive pathogenic one (an antagomir) could, in principle, act on several pathways at once (Lorente-Cebrián et al., 2019). However, most studies show biological relevance rather than therapeutic efficacy. For example, Castaño et al. found that exosomes loaded with miR-122, miR-192, and miR-27 mimics induced glucose intolerance, insulin resistance, and hepatic steatosis when administered to healthy mice, identifying these miRNAs as pathogenic drivers and candidate antagomir targets (Castaño et al., 2018). Conversely, Ji et al. demonstrated that an antagomir against miR-3075 worsened glucose tolerance *in vivo*, confirming its necessity for normal insulin sensitivity (Ji et al., 2021). However, the corresponding mimic was tested only in cultured cells and primary hepatocytes, so its therapeutic efficacy remains unknown.

Similar limitations apply to other emerging RNA classes. circRNAs can act as molecular sponges that sequester specific miRNAs away from their normal mRNA targets, and ceRNA network analyses have linked one such circRNA, circGhr, to weight-loss responses in liraglutide-treated obese mice (Li W. et al., 2025), but such mechanistic and correlative findings are preliminary; studies conducting direct *in vivo* circRNA manipulation with readouts of therapeutic efficacy are required to present translational potential.

Evidence for tRF-targeted therapies is beginning to emerge, although they remain largely preclinical. In pancreatic β-cell models, antisense inhibition of the tRF 5′tRFGlu (CTC) improved cell survival under lipotoxic stress, reduced ER stress, preserved insulin secretion, and limited the pro-inflammatory macrophage response that exacerbates β-cell injury (Cosentino et al., 2026). Separately, tRFGlu-TTC has been implicated in adipose biology, where it promotes preadipocyte proliferation and inhibits differentiation through regulation of KLF9, KLF11, and KLF12 (Pan et al., 2021). These findings suggest that tRFs may represent tractable therapeutic targets in both diabetes and obesity. However, as with circRNA-based approaches, convincing *in vivo* evidence of therapeutic efficacy remains limited.

Aptamers occupy a distinct position because they can act as both targeting ligands and therapeutic agents. These short single-stranded nucleic acids are selected *in vitro*, via SELEX-based approaches, for their ability to fold into shapes that bind a target with antibody-like specificity (Li Y. et al., 2025). Adipo8 and its bivalent derivative Adipo8cB bind directly to mature adipocytes and improve metabolic health in obese mice by reducing adiposity, improving glucose tolerance, and limiting hepatic steatosis (Zhang et al., 2024). However, much of the adipose-targeting aptamer literature is based on a single platform, including several subsequent delivery systems built on the same scaffold (Chen et al., 2022; Wang et al., 2026). As a result, independent validation remains limited, making it difficult to assess the generalisability of these findings.

CRISPR/Cas9-based gene editing offers an alternative to siRNA/ASO knockdown, capable of producing durable changes in gene expression. Targeted CRISPR interference against Fabp4 in white adipocytes, delivered via a peptide-mediated non-viral system, reduced inflammatory cytokines, improved insulin sensitivity, and achieved a 20% reduction in body weight in obese mice (Chung et al., 2019). Separately, CRISPR-SAM-mediated activation of UCP1 in engineered human brown-like adipocytes induced thermogenesis and improved glucose tolerance after transplantation into obese mice (Wang C-H. et al., 2020). However, the Fabp4 study’s reliance on a prohibitin-targeting peptide carries the same off-target concerns raised earlier for prohibitin-based siRNA delivery.

Taken together, the field has firmly established that RNA silencing works and that nanoparticle cargoes can be made flexible; what it has not established is a reproducible, receptor- or anatomy-based route specifically into adipose or visceral tissue. Delivery specificity is the major constraint on the success of an RNA therapeutic, which is why aptamers and anatomy-exploiting platforms like Lei et al.'s are appealing, since neither depends on an adipose-specific receptor (Le et al., 2026).

## 5 Challenges and limitations

Many of the limitations facing RNA research in obesity are not specific to any one RNA class but rather structural features of the field. Across both biomarker and therapeutic studies, the recurring issues include small single-centre cohorts, overinterpretation of mechanistic findings as biomarker evidence, and failure to demonstrate added value beyond established clinical measures. Correlational associations are frequently presented as evidence of causality, while short follow-up periods make it difficult to determine whether observed RNA changes reflect obesity itself, associated comorbidities, or longer-term disease trajectories. For instance, several obesity-associated lncRNAs remain elevated in obesity and fail to normalise following weight loss, leaving their significance unresolved (Sun et al., 2016). Short follow-up also limits the evaluation of long-term safety and durability of therapeutic intervention.

Additional challenges are specific to RNA therapeutics. Tissue-specific EVs represent only a small fraction of circulating vesicles (Shami-shah et al., 2023), complicating attribution of biological effects to a particular organ of origin. Delivery efficiency remains a major bottleneck: only a small fraction of nucleic acid cargo successfully escapes the endosome into the cytosol upon LNP-triggered membrane damage, with many damaged endosomes containing no detectable cargo at all (Johansson et al., 2025), and the mechanisms governing this process remain poorly understood. Progress has been further hindered by the lack of nanoscale-resolution imaging techniques capable of visualising endosomal escape with sufficient spatial and temporal resolution (Chatterjee et al., 2024). Finally, poor sequence conservation of many ncRNAs across species limits the reliability of rodent models (Sarropoulos et al., 2019; Lluch et al., 2024), and the heterogeneity of obesity, driven by interacting genetic, environmental, and lifestyle factors, reduces the likelihood that universal RNA signatures or therapeutic targets will emerge (Iacomino et al., 2019).

## 6 Conclusion and perspectives

Advances in RNA biology have moved RNA-based strategies for obesity beyond theoretical promise, with preliminary clinical findings from ARO-INHBE and ARO-ALK7 (Arrowhead Pharmaceuticals Inc, 2026) providing early evidence of RNA-mediated therapeutic activity in hepatic and adipose pathways, though both require peer-reviewed validation before firm conclusions are drawn.

For biomarkers, progress requires prospective validation of the strongest existing candidates against established clinical tools, with large longitudinal cohorts sufficient to establish robust diagnostic thresholds across disease stages and between individuals. Candidate RNA biomarkers must be evaluated in studies that account for environmental and lifestyle factors known to influence RNA expression and formally tested against routine clinical measures to assess incremental diagnostic or prognostic value.

For therapeutics, the central unresolved problem is extrahepatic delivery. The GalNAc-ASGPR system demonstrates that receptor-based hepatic targeting is clinically achievable; replicating this specificity in adipose tissue, where no validated surface marker exists, remains the field’s most important technical challenge. Anatomy-exploiting platforms and aptamer-based dual-function strategies are encouraging, but neither has produced reproducible, scalable evidence in metabolically relevant human depots. Improving long-term safety and durability data, which most preclinical designs do not provide, is equally necessary for translation.

The broader aim of RNA-based intervention, moving beyond symptomatic management towards mechanism-driven targeting of upstream metabolic pathways, is scientifically well-motivated. Whether it is achievable depends on whether the field is willing to prioritise rigorous validation and delivery engineering over the continued production of early-stage mechanistic findings.
